# A single unified model for fitting simple to complex receptor response data

**DOI:** 10.1038/s41598-020-70220-w

**Published:** 2020-08-07

**Authors:** Peter Buchwald

**Affiliations:** Department of Molecular and Cellular Pharmacology and Diabetes Research Institute, Miller School of Medicine, University of Miami, Miami, FL 33136 USA

**Keywords:** Pharmacodynamics, Receptor pharmacology, Applied mathematics

## Abstract

The fitting of complex receptor-response data where fractional response and occupancy do not match is challenging. They encompass important cases including (a) the presence of “receptor reserve” and/or partial agonism, (b) multiple responses assessed at different vantage points along a pathway, (c) responses that are different along diverging downstream pathways (biased agonism), and (d) constitutive activity. For these, simple models such as the well-known Clark or Hill equations cannot be used. Those that can, such as the operational (Black&Leff) model, do not provide a unified approach, have multiple nonintuitive parameters that are challenging to fit in well-defined manner, have difficulties incorporating binding data, and cannot be reduced or connected to simpler forms. We have recently introduced a quantitative receptor model (SABRE) that includes parameters for Signal Amplification (*γ*), Binding affinity (*K*_d_), Receptor activation Efficacy (*ε*), and constitutive activity (*ε*_R0_). It provides a single equation to fit complex cases within a full two-state framework with the possibility of incorporating receptor occupancy data (i.e., experimental *K*_d_s). Simpler cases can be fit by using consecutively reduced forms obtained by constraining parameters to specific values, e.g., *ε*_R0_ = 0: no constitutive activity, *γ* = 1: no amplification (*E*_max_-type fitting), and *ε* = 1: no partial agonism (Clark equation). Here, a Hill-type extension is introduced (*n* ≠ 1), and simulated and experimental receptor-response data from simple to increasingly complex cases are fitted within the unified framework of SABRE with differently constrained parameters.

## Introduction

Receptors^1,2^ lie at the core of pharmacology and our current mechanism of drug action theories^3–5^. It is now well understood that xenobiotics can generate physiological effects depending on (1) the amount of active compound that actually reaches some receptor or, in more general terms, an “effect” compartment and (2a) the strength of the interaction and (2b) the relevance of the structural changes produced at this site. Whereas, the former are determined by processes related to the pharmacokinetic (PK) phase (including absorption, distribution, metabolism, and elimination; ADME), the latter are determined by processes related to the pharmacodynamic (PD) phase (including the ability to bind and activate a receptor, i.e., affinity and efficacy, respectively). Regarding the latter, our sole focus here, it is now well recognized that in order to accommodate phenomena such as partial agonism and receptor reserve, where fractional responses lag behind or are ahead of fractional occupancy, receptor models have to be two-state models in which ligand-occupied receptor states are not necessarily active (response-generating) and vice versa, active receptor states are not necessarily ligand occupied. Associating such two-state models with quantitative receptor models to establish general concentration–response (or, more generally, dose- or exposure–response) relationships is crucial, as “exposure–response information is at the heart of any determination of the safety and effectiveness of drugs”^6^ and quality pharmacological analysis should always include rigorous quantitative analyses and the calculation and reporting of IC_50_, EC_50_, *K*_*d*_, and other such values.

**Figure 1:**
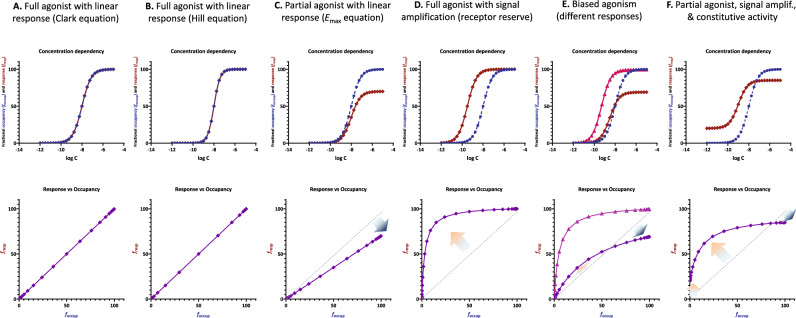
Relationship between fractional response (*f*_resp_ = *E*/*E*_max_) and occupancy (*f*_occup_ = [LR_occup_]/[LR_max_]) for various increasingly complex assumptions (**A**–**F**) as shown by the titles on top and discussed in detail in the text. Top row: receptor response (red) and occupancy (blue) as a function of ligand concentration on typical semilog scales (*f*_resp_ and *f*_occup_ as a function of log *C* = log [L]). For the simplest cases (**A**-**B**), they overlap, and the red line is not visible. Bottom row: corresponding response versus occupancy curves (*f*_resp_ as a function of *f*_occup_). Note that in some of the more complex cases, the fractional response can be both ahead and behind the fractional occupancy even for the same compound (red and blue arrows indicating deviations from the unity line in **E** and **F**). While these are simulated data, experimental data illustrating such cases are also available (see, e.g., references^11–14^ and discussions in^15,16^).

Well-known important cases where fractional response and occupancy are not aligned and challenging to fit include (a) the presence of “receptor reserve” (“spare receptors”)^7^ and/or partial agonism (often in combination)^8,9^, (b) multiple responses assessed at different vantage points along a signaling pathway, (c) responses that are different along distinct divergent downstream pathways originating from the same receptor (biased agonism, functional selectivity), and (d) constitutive activity. A set of illustrative examples are included in Fig. 1 showing the concentration-dependency of occupancy and response (top row) as well as the corresponding response versus occupancy curves (bottom row). In cases **D**–**F**, the response curves (red) are left-shifted compared to occupancy (blue) due to signal amplification; cases where they are right-shifted are less common and are usually indications of an occupancy threshold issue (i.e., receptor concentration not being negligible compared to that of the ligand)^10^.

**Figure 2:**
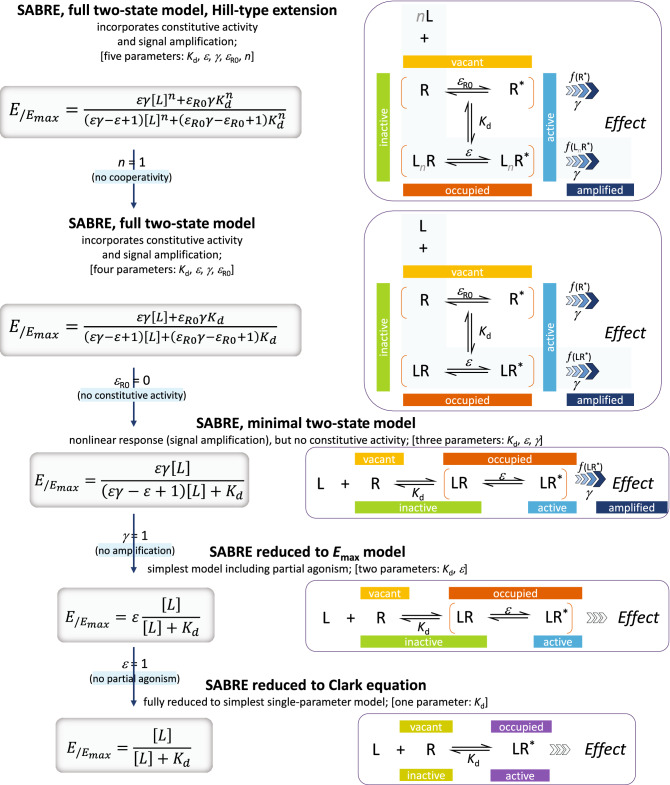
The present SABRE receptor model in its most general full two-state form with Hill-type extension (top) and its consecutively nested simplifications down to the Clark equation (bottom). For each case, the corresponding quantitative form connecting fractional response (*f*_resp_ = *E*/*E*_max_) to ligand concentration [L] is shown at left and a schematic illustration of receptor binding and activation at right.

For the more complex cases (such as those in Fig. 1C–F), the well-known and straightforward Clark equation

1 $${E}_{/{E}_{max}}={f}_{resp}=\frac{\left[L\right]}{\left[L\right]+{K}_{d}}$$

and its Hill-type extension

2 $${E}_{/{E}_{max}}={f}_{resp}=\frac{{\left[L\right]}^{n}}{{\left[L\right]}^{n}+{K}_{d}^{n}}$$

cannot be used as they assume responses proportional with occupancy; hence, do not allow separation between fractional response, *f*_resp_ = *E*_/*E*max_, and occupancy, *f*_occup_ = [LR_occup_]/[LR_max_] (Fig. 1A,B). They do not include parametrization for efficacy, only for occupancy via *K*_d_, the classic equilibrium dissociation constant characterizing receptor binding, which is defined in terms of the concentrations of the species involved (see Fig. 2, bottom row) and hence measured in units of concentrations:

3 $${K}_{d}=\frac{\left[L\right]\left[R\right]}{\left[{LR}^{*}\right]}$$

In most of these more complex cases, one can use the more cumbersome operational (Black & Leff) model (Eq. 4)^17^ or its variations for fitting response data because it has an additional efficacy type parameter (*τ*):

4 $${E}_{/{E}_{max}}={f}_{resp}=\frac{\tau \left[L\right]}{\left(\tau +1\right)\left[L\right]+{K}_{D}}$$

However, this model in its strict two-parameter version cannot connect response to occupancy because it cannot incorporate experimental binding affinities since its *K*_D_ parameter is a fitted one that is not related to the experimental dissociation constant, *K*_d_. The discrepancy is especially serious for full or close to full agonists, where *τ* needs to have large values^16^. Related to this, an important limitation of the operational model is that most of the time, it is difficult to fit in a non-ambiguous manner, i.e., to obtain well-defined parameters. If just functional data are available (i.e., a single concentration–response curve), only the so-called transduction coefficient (*τ*/*K*_D_) and not *K*_D_ and *τ* independently can be estimated precisely due to identifiability issues during regression^18,19^.

To overcome these limitations, we have recently introduced a quantitative receptor model (SABRE) that can fit all these cases of increasing complexity with a unified equation using parameters for Signal Amplification (*γ*), Binding affinity (*K*_d_), and Receptor activation Efficacy (*ε*) (plus constitutive activity, *ε*_R0_, if needed)^15,16^. Its most general form that also includes a Hill coefficient (*n*), which will be derived here, is:

5 $${E}_{/{E}_{max}}=\frac{\varepsilon \gamma {\left[L\right]}^{n}+{\varepsilon }_{R0}\gamma {K}_{d}^{n}}{\left(\varepsilon \gamma -\varepsilon +1\right){\left[L\right]}^{n}+\left({\varepsilon }_{R0}\gamma -{\varepsilon }_{R0}+1\right){K}_{d}^{n}}$$

As explicit incorporation of a signal amplification parameter is a main novelty, the model was designated SABRE as highlighted by the capitalization above. Not only is SABRE using parameters that are more intuitive and easier to interpret than those of the operational model or the del Castillo-Katz minimal two-state model, but, contrary to those, it can fit both simple and complex cases with the same equation (Eq. 5) with differently constrained parameters. It can be collapsed into consecutive simplified forms by fixing its parameters as special values (Fig. 2), and these can and should be used on their own when adequate. By introducing independent parametrization for the (post-receptor) signal amplification, SABRE allows a more clear conceptualization of receptor signaling as separate processes of binding, activation, and signal transduction (amplification) that can now be characterized and quantified via their own distinct parameters: *K*_d_, *ε*, and *γ*, respectively (Fig. 2).

Here, receptor response data of increasing complexity were fitted with SABRE using different levels of parameter constraining to illustrate the advantage of a unified model that allows nested simplifications (Fig. 2). Main details of model parametrization (including its Hill-type extension) are summarized in the Method section below followed by illustrative fittings of simulated and experimental response versus occupancy data in the Results section. These range from the simplest case, *E*_max_-type response only data, to complex multiple responses and biased agonism examples.

## Methods

### Model concepts

The present model maintains the main assumptions of the two-state receptor theory, e.g., that ligand-bound (occupied) and active receptor states do not fully correspond but introduces a slightly different and more intuitive parametrization—a detailed discussion is included in Ref.^16^. Briefly, binding of the ligand is assumed to alter the likelihood of activation: receptors can be active or inactive in both their ligand-free and ligand-bound forms (Fig. 2, top right); however, the corresponding probabilities (i.e., times spent in the respective conformations) can be quite different. Hence, ligand-free (R) and ligand-bound (LR) states are considered as an equilibrium ensemble of active (^*^) and inactive conformations present: R ⇌ R^*^ and LR ⇌ LR^*^, respectively (not excluding the possibility that multiple, possibly overlapping active states might exist). In general, ligand-free receptors are overwhelmingly in their inactive conformation, R. In cases where there is no constitutive activity, they are entirely so. Binding of an agonist, which is governed by the affinity parameter *K*_d_, shifts the equilibrium toward the active state. The ability of a bound ligand to do so is characterized by an (intrinsic) efficacy parameter, *ε*. For receptors with constitutive activity, a basal receptor efficacy, *ε*_R0_, is used to account for baseline activation even in absence of a ligand. The signal (response) generated by the active receptor (R^*^ or LR^*^) can be amplified downstream, and this is characterized by a pathway-specific gain parameter *γ*. Hence, the model uses four parameters: *K*_d_, the equilibrium dissociation constant characterizing binding affinity; *ε*, the efficacy characterizing the ability of bound ligand to activate the receptor (0 ≤ *ε* ≤ 1); *ε*_R0_, a basal receptor efficacy characterizing constitutive activity (0 ≤ *ε*_R0_ ≤ 1); and *γ*, a gain (amplification) parameter characterizing the nonlinearity of (post-activation) signal transduction (1 ≤ *γ*). To accommodate responses that are more (or less) abrupt than those corresponding to a straightforward law of mass action, an additional a Hill-coefficient parameter, *n*, is also introduced here. Corresponding equations, schematics, and all successive nested simplifications leading to special cases are summarized in Fig. 2.

### Parametrization

Occupancy (binding) parametrization, is achieved via a *K*_d_ parameter similar to that of the original simple definition (Eq. 3), but with some modifications. SABRE differentiates between active and inactive receptor states (denoted by an asterisk, i.e., R^*^ vs R and LR^*^ vs LR), but considers ligand-bound and ligand-free states as an ensemble of conformations, so that *K*_d_ represents an average binding constant for these ensembles of active and inactive forms that the ligand effectively sees. Hence, *K*_d_ is defined in terms of the overall concentrations of occupied and unoccupied (ligand-free) receptors (Fig. 2A)

6 $${K}_{d}=\frac{\left[L\right]\left[{R}_{free}\right]}{\left[{R}_{occup}\right]}=\frac{\left[L\right]\left(\left[R\right]+\left[{R}^{*}\right]\right)}{\left(\left[LR\right]+\left[{LR}^{*}\right]\right)}$$

Accordingly, SABRE does not distinguish between binding affinities for the active and inactive states (as done by other two-state models, e.g., *K*_d_ and *K*_d_/*α*;^16^). It uses a single, ensemble-averaged *K*_d_ that is a macroscopic equilibrium constant and, hence, experimentally measurable in equilibrium binding assays that assess total binding to the receptor. While this deviates from the assumptions of other models, the validity of such a single, experimentally measurable binding constant is nicely supported by previous works quantifying binding via both static (*K*_d_) and kinetic (*k*_off_/*k*_on_) methods in parallel with multiple downstream effects that found the experimental binding constants derived by these two methods to be in very close agreement (e.g., for the M_3_ muscarinic receptor^12^ and for the μ-opioid receptor^20^).

Efficacy parametrization here is achieved via an *ε* parameter that represents the fraction of ligand-bound receptors that are active^15,16^:

7 $$\varepsilon =\frac{\left[L{R}^{*}\right]}{\left[{LR}_{tot}\right]}=\frac{\left[{LR}^{*}\right]}{\left[LR\right]+\left[L{R}^{*}\right]}$$

Hence, *ε* is a unitless parameter in the 0 to 1 range, and represents an intrinsic efficacy measured immediately post-receptor. Note, however, that it is not an equilibrium constant such as *K*_ε_ = [LR^*^]/[LR]). Constitutively active receptors are incorporated into the formalism of SABRE via a baseline receptor efficacy (*ε*_R0_) that is defined along similar lines, but for ligand-free receptors (i.e., the fraction of ligand-free receptors that are active):

8 $${\varepsilon }_{{R}_{0}}=\frac{\left[{R}^{*}\right]}{\left[{R}_{tot}\right]}=\frac{\left[{R}^{*}\right]}{\left[R\right]+\left[{R}^{*}\right]}$$

While *ε* is a ligand characteristic, *ε*_R0_ is a receptor characteristic. With these definitions, inverse agonists that reduce the signaling output below that of the basal state will have *ε* < *ε*_R0_. Full agonists have *ε* = 1; partial agonists that generate a response but cannot reach the maximum one even at concentrations that saturate all receptor sites have *ε*_R0_ < *ε* < 1, and neutral antagonists have *ε* = *ε*_R0_.

Finally, the present model explicitly incorporates pathway-specific signal transduction (amplification) via a separate gain parameter *γ*. Signal amplification has to be built into receptor models to account for cases where almost maximal responses can be achieved at relatively small fractional occupancies, i.e., cases that were traditionally designated as having “receptor reserve” or “spare receptors”. In SABRE, the fraction of active receptors

9 $${f}_{act}=\frac{\left[{R}^{*}\right]+\left[{LR}^{*}\right]}{\left[{R}_{tot}\right]}=\frac{\left[{R}^{*}\right]+\left[{LR}^{*}\right]}{\left[R\right]+\left[{R}^{*}\right]+\left[LR\right]+\left[{LR}^{*}\right]}=\frac{{\varepsilon }_{R0}{K}_{d}+\varepsilon \left[L\right]}{\left[L\right]+{K}_{d}}$$

is linked to the fractional response, *f*_resp_ = *E*_/*E*max_, via a hyperbolic function. Such functions provide convenient ways to incorporate signal amplification cascades and have been shown to exist in response vs occupancy data. However, in SABRE not *f*_act_ itself, but its odds-ratio type transform

10 $$\Lambda =\frac{{f}_{act}}{1-{f}_{act}}$$

serves as input (see Ref.^15^ for details):

11 $${f}_{resp}={E}_{{/E}_{max}}=\frac{\Lambda }{\Lambda +{\gamma }^{-1}}; \Lambda =\frac{{f}_{act}}{1-{f}_{act}}$$

With this definition, *γ* represents a unitless amplification (gain) factor (*γ* ≥ 1). After some transformations, this results in the final form of the full four-parameter model linking fractional response, *f*_resp_ = *E*/*E*_max_, to ligand concentration [L]:

12 $${E}_{/{E}_{max}}=\frac{\varepsilon \gamma \left[L\right]+{\varepsilon }_{R0}{\gamma K}_{d}}{\left(\varepsilon \gamma -\varepsilon +1\right)\left[L\right]+{\left({\varepsilon }_{R0}\gamma -{\varepsilon }_{R0}+1\right)K}_{d}}$$

For cases with no constitutive activity (*ε*_R0_ = 0; no active unbound receptor, R^*^), this reduces to the three-parameter minimal two-state model previously introduced^15^ (third row of Fig. 2) and the simplified equation:

13 $${E}_{/{E}_{max}}=\frac{\varepsilon \gamma \left[L\right]}{\left(\varepsilon \gamma -\varepsilon +1\right)\left[L\right]+{K}_{d}}$$

A detailed derivation of these equations, including its generalization for a Hill-type extension, is included in Supporting Information, Appendix 1. A better interpretation of these parameters can be gleaned from a slightly rearranged form of this three-parameter equation, which corresponds to a case with Hill coefficient slope of unity (*n* = 1):

14 $${E}_{/{E}_{max}}=\frac{\varepsilon \gamma }{\left(\varepsilon \gamma -\varepsilon +1\right)}\frac{\left[L\right]}{\left[L\right]+\frac{{K}_{d}}{\left(\varepsilon \gamma -\varepsilon +1\right)}}$$

From here, it is clear that half-maximal response (EC_50_) is observed at *K*_obs_ = *K*_d_/(*εγ*–*ε* + 1) and the maximum (fractional) effect achievable by a given ligand is *f*_resp,max_ = *εγ*/(*εγ*–*ε* + 1). For full agonists at the receptor (*ε* = 1), *K*_obs_ = *K*_d_/*γ*; therefore, the gain *γ* is a straightforward multiplication factor causing a left shift of the sigmoid response function by *γ* units on a semi-log scale. By explicitly separating the parametrization of pathway-specific amplification (*γ*) from that of ligand-specific receptor activation (*ε*), SABRE more clearly outlines than other models the *intrinsic efficacy* concept, which proved to be somewhat elusive in pharmacology^21,22^.

### Hill-type extension (cooperative binding)

As a final step, a Hill-type parametrization can also be introduced via a Hill slope, *n*, to allow either more (*n* > 1) or less (*n* < 1) abrupt concentration-dependent responses:

15 $${E}_{/{E}_{max}}=\frac{{\left[L\right]}^{n}}{{\left[L\right]}^{n}+{K}_{d}^{n}}$$

This function, originally introduced by Hill based on empirical considerations^23^, provides a versatile mathematical function often used in pharmacological^24,25^ and other applications^26^. It shows analogy with the logistic function, one of the most widely used sigmoid functional forms, as it is equivalent with a logarithmic logistic function, *y* = *f*(*x*) = *R*_max_/(1 + *βe*^–*n*ln*x*^)^27^. In fact, the classic sigmoid shapes obtained in typical semi-log scale graphs (such as those shown in Fig. 1) are those of the logistic function. The IUPHAR recommendation is to use “Hill equation” for the relationship between ligand concentration and effect (such as Eq. 15 here), whereas “Hill–Langmuir equation” should be used for relationship between ligand concentration and occupancy^27^. Hill slopes different from unity are typically indications of interacting binding sites with positive (*n* > 1) or negative (*n* < 1) cooperativity^28^. The Clark equation (Eq. 1), as well as the analogous Michaelis–Menten equation, represent a special case (*n* = 1) of the Hill equation—a nice example of how more complex models can be collapsed into simplified forms for special cases of their parameters.

Following a detailed derivation (see Supporting Information, Appendix 1), the Hill-type extension of the general form of the present SABRE model that includes constitutive activity is a straightforward generalization of Eq. 12 introducing the Hill-coefficients as exponents of the concentration terms:

16 $${E}_{/{E}_{max}}=\frac{\varepsilon \gamma {\left[L\right]}^{n}+{\varepsilon }_{R0}\gamma {K}_{d}^{n}}{\left(\varepsilon \gamma -\varepsilon +1\right){\left[L\right]}^{n}+\left({\varepsilon }_{R0}\gamma -{\varepsilon }_{R0}+1\right){K}_{d}^{n}}$$

In case of no constitutive activity, this simplifies to the Hill-type extension of Eq. 13:

17 $${E}_{{/E}_{max}}=\frac{\varepsilon \gamma {\left[L\right]}^{n}}{\left(\varepsilon \gamma -\varepsilon +1\right){\left[L\right]}^{n}+{K}_{d}^{n}}$$

### Consecutive simplifications

An important feature of the present model is that, contrary to previous quantitative receptor models such as those based on the operational model, its general form (Eq. 5) can be reduced to consecutively nested simplified forms for special cases of its parameters (Fig. 2), and these can be used on their own when adequate. Hence, the very same model can be used to fit data of various complexity levels using different sets of constrained parameters. First, by setting *n* = 1, it reproduces the simpler form of SABRE as introduced before^16^, just as setting *n* = 1 in the Hill equation (Eq. 2 or 15) reproduces the Clark equation (Eq. 1) as its simpler form.

18 $${E}_{/{E}_{max}}=\frac{\varepsilon \gamma {\left[L\right]}^{1}+{\varepsilon }_{R0}\gamma {K}_{d}^{1}}{\left(\varepsilon \gamma -\varepsilon +1\right){\left[L\right]}^{1}+\left({\varepsilon }_{R0}\gamma -{\varepsilon }_{R0}+1\right){K}_{d}^{1}}=\frac{\varepsilon \gamma \left[L\right]+{\varepsilon }_{R0}{\gamma K}_{d}}{\left(\varepsilon \gamma -\varepsilon +1\right)\left[L\right]+{\left({\varepsilon }_{R0}\gamma -{\varepsilon }_{R0}+1\right)K}_{d}}$$

The way the Hill equation can be reduced to the Clark equation by constraining one of its parameters to a special value (*n* = 1) has been a long-known example in quantitative pharmacology. With SABRE, one can do the same and more, as one can continue with multiple consecutive simplifications of its parameters. Cases with no constitutive activity (no R^*^ form) can be obtained by constraining *ε*_R0_ to 0 leading to the three-parameter minimal two-state SABRE model that was the first form introduced^15^:

19 $${E}_{/{E}_{max}}=\frac{\varepsilon \gamma \left[L\right]+0\gamma {K}_{d}}{\left(\varepsilon \gamma -\varepsilon +1\right)\left[L\right]+{\left(0\gamma -0+1\right)K}_{d}}=\frac{\varepsilon \gamma \left[L\right]}{\left(\varepsilon \gamma -\varepsilon +1\right)\left[L\right]+{K}_{d}}$$

Further, if there is no amplification (or if it cannot be reliably evaluated due to lack of independently assessed occupancy/binding data), *γ* should be constrained to 1, and the model collapses further into the simple two-parameter *E*_max_ model of partial agonism (Fig. 2):

20 $${E}_{/{E}_{max}}=\frac{\varepsilon 1\left[L\right]}{\left(\varepsilon 1-\varepsilon +1\right)\left[L\right]+{K}_{d}}=\frac{\varepsilon \left[L\right]}{\left[L\right]+{K}_{d}}$$

Finally, if there is no partial agonism (i.e., all occupied receptors are active), *ε* can be constrained to 1, and this leads to the simple one-parameter Clark equation (Fig. 2, bottom row):

21 $${E}_{/{E}_{max}}=\frac{1\left[L\right]}{\left[L\right]+{K}_{d}}$$

Regarding these consecutively nested simplified forms, it is important to always use the simplest one (i.e., the one with the largest number of fixed parameters) that still provides adequate fit to limit the number of adjustable parameters^29–32^. Adequate fitting requires the availability of 5–10 (well-distributed) data points for each adjustable parameter^5,33,34^; hence, reliable fitting of the full model can only be accomplished if sufficiently large number of data points are available. Because of its separate amplification parameter, SABRE uses one more parameter than the operational model, e.g., three (*K*_d_, *ε*, *γ*) versus two (*K*_d_, *τ*) for the case of no constitutive activity (Eq. 19 vs. 4) or four (*K*_d_, *ε*, *γ, ε*_R0_; Eq. 18) versus three (e.g., *K*_d_, *ε*, *χ*; Eq. 22) for cases with constitutive activity—if comparing with the Slack and Hall version of the extended operational model shown below^35–37^:

22 $${E}_{/{E}_{max}}=\frac{\chi \left({K}_{D}+\varepsilon \left[L\right]\right)}{\left({K}_{D}+\left[L\right]\right)+\chi \left({K}_{D}+\varepsilon \left[L\right]\right)}; \chi =\frac{\left[{R}_{tot}\right]}{{K}_{\varepsilon }}$$

However, the need for one extra parameter is more than compensated for by (1) the more intuitive nature of the present parameters (due to their more clear connection to receptor binding, activation efficacy, and signal amplification), and (2) the ability to use simplified forms with constrained parameters so that fewer need fitting. Contrary to the operational model and its variations, SABRE can be reduced to simplified forms for special cases of its parameters, and these can and should be used on their own when adequate or when there are not enough data to support full parametrization.

### Implementation and data fitting

All data are normalized and have no baseline (i.e., in the 0–100% range). Data fittings were performed in GraphPad Prism (GraphPad, La Jolla, CA, USA, RRID:SCR_002798). Fitting with the present model were done with a custom implementation corresponding to the general Eq. 5, which is available for download (Supporting Information) and with parameters constrained as indicated for each case. Simulated data were generated with the same model in Prism using the “Simulate XY data” algorithm with 5% random error). Experimental data used for illustrations of model fit are reproduced from previous publications as indicated in the corresponding figures.

## Results and discussion

In systems with signal amplification, ligands of different efficacies (e.g., full, partial, and possibly inverse agonist) can produce complex concentration–response functions leading to complicated connections between fractional response (*f*_resp_) and occupancy (*f*_occup_) (Fig. 1) that can be fit only by multi-parameter models. So far, no single quantitative receptor model could fit simple as well as complex cases within a unified framework. Here, it is shown how this can be done with the present model, which allows consecutive nested simplifications, using a set of examples of increasing complexity. Simulated as well as experimental data were fitted with the same general equation (Eq. 5), but with different levels of parameter constraining. To avoid excessive parametrization, all cases assume *n* = 1, i.e., no Hill-type extension (except for one specific illustration in Fig. 4), as well as *ε*_R0_ = 0 (no constitutive activity).

### Response only data (***E***_max_ model with possible Hill-type extension)

**Figure 3:**
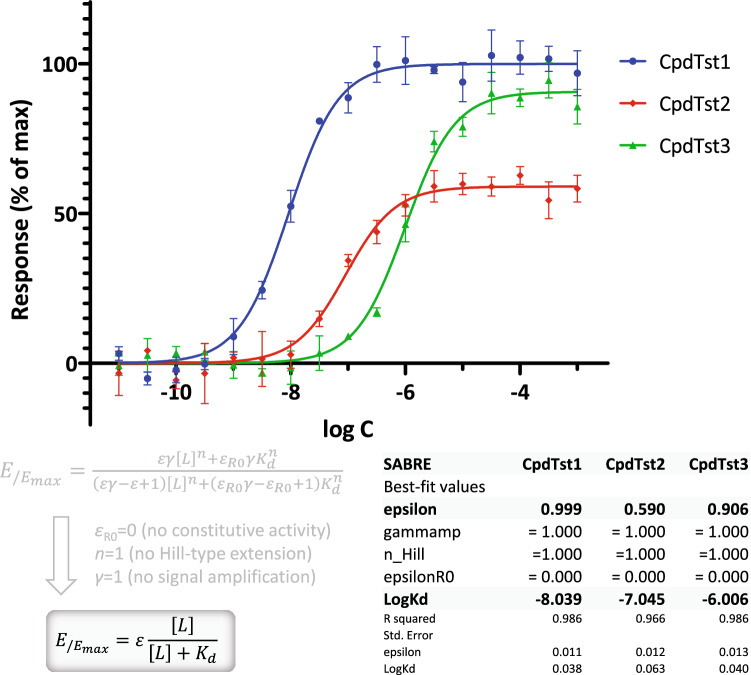
Fit of response only (EC_50_) data (full and partial agonists) with the present SABRE model. Simulated data (symbols) for three different compounds were generated in GraphPad Prism (“Simulate XY data” algorithm with 5% random error) and then fitted with SABRE (lines) using the constrained version as shown (Eq. 5; *ε*_R0_ = 0, *γ* = 1, *n* = 1). This is equivalent with fit with the *E*_max_ model, e.g., the widely used “log(agonist) vs. response (three parameters)” model, and results in exactly the same parameters (assuming a 0 baseline).

As the simplest first case, let us consider the fitting of plain response data to determine EC_50_ (*K*_obs_) values for agonists of different potencies in a given assay system. EC_50_ is the half-maximal (or median) effective concentration (or dose, ED_50_), which is the concentration (dose) of an agonist that produces 50% of the maximum possible activity of that agonist^27^. Such EC_50_ determinations are routinely done in pharmacology using either the one-parameter Clark equation (if only full agonists are present) or the two-parameter *E*_max_ equation (if partial agonists are also present). These models are widely used, familiar to those performing such nonlinear regression-based fit, and implemented in a variety of software packages, e.g., “log(agonist) vs. normalized response” and “log(agonist) vs. response (three parameters)” models in GraphPad Prism. The same fit can be obtained with SABRE (Eq. 5) by constraining all but one (*K*_d_) or two (*K*_d_, *ε*) of its parameters. An illustrative example using simulated data for three different hypothetical agonists is shown in Fig. 3. Fit with SABRE reproduced perfectly both the log *K*_d_ values (which here correspond to log EC_50_s; − 6, − 7, and − 8) and maximum fractional response (efficacy) values (100%, 90%, and 60%) used to generate the data. To have realistic data with some scatter, a 5% random error was allowed. Hence by restricting its parameters, SABRE can be reduced to reproduce simpler forms that can be used on their own, something that cannot be done with models based on the operational model.

**Figure 4:**
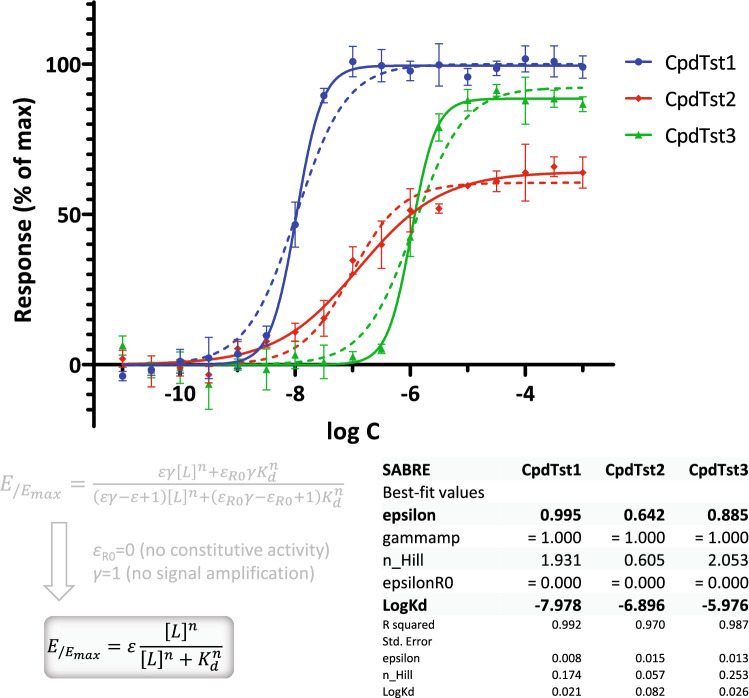
Fit of response only (EC_50_) data for cases with non-unity Hill slopes. Simulated data (symbols) generated as before (Fig. 3) but with *n* ≠ 1 and then fitted with SABRE (lines) using the constrained version as shown (Eq. 5; *ε*_R0_ = 0, *γ* = 1). To illustrate the effect of releasing *n*, fit with constrained unity slopes are also shown (*n* = 1, dashed lines)—note that fit is considerably improved, but EC_50_s are essentially unaffected.

If response data are either more or less abrupt than predicted by the standard law of mass action (unity Hill slope, *n* = 1), the Hill coefficient of Eq. 5 also needs to be released resulting in three-parameter fit (*K*_d_, *ε*, *n*). An illustration of this is shown in Fig. 4 with data generated as in Fig. 3 but with Hill slopes of 2.0 and 0.66. In most cases, *n* should be constrained to the same value for all compounds assessed in the same assay; here, this was not done to illustrate the effect of both *n* > 1 and *n* < 1.

### Connecting response to independently measured occupancy data

**Figure 5:**
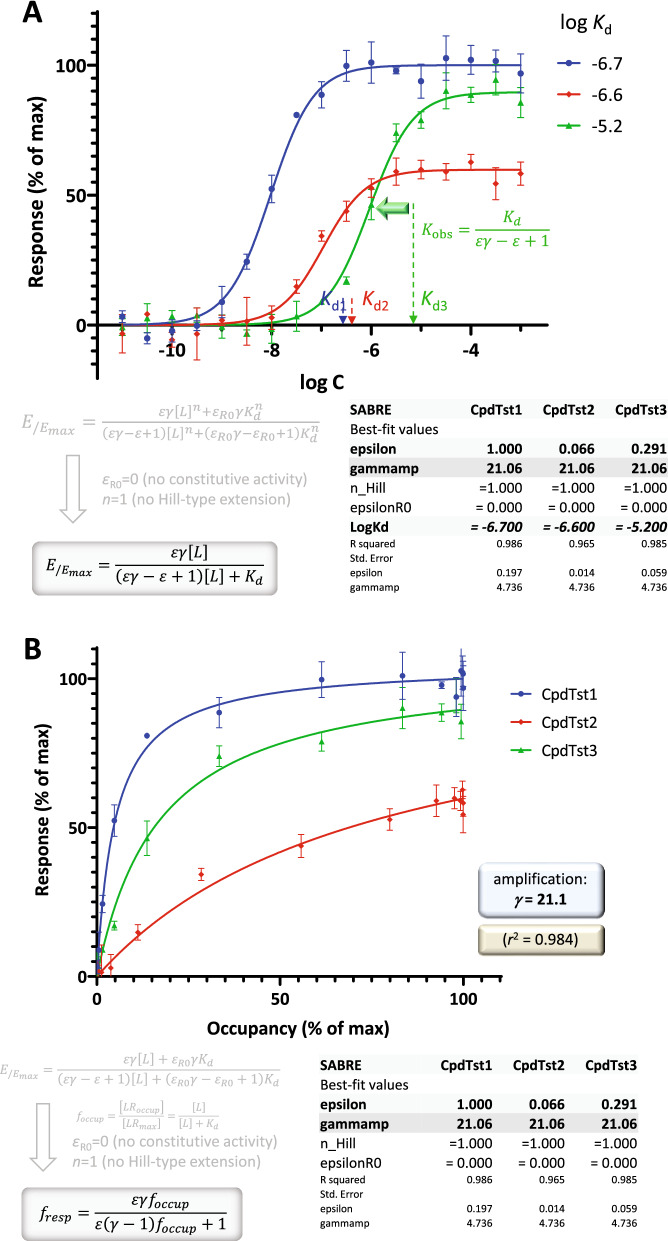
Fit of independently measured response (EC_50_) and occupancy (*K*_d_) data with the present SABRE model. (**A**) Concentration–response data that are the same as in Fig. 3, but were fitted here with incorporation of an additional set of *K*_d_s. The corresponding parameters were restricted as shown (log *K*_d_s of − 6.7, − 6.6, and − 5.2; indicated on the *x* axis), and only one *γ* and three *ε*s were fitted (*n*_p_ = 4). (**B**) Direct fit of corresponding response versus occupancy data. Same data as **A**, but fitting done directly with Eq. 23 linking *f*_resp_ to *f*_occup_. If *f*_occup_ is calculated from *K*_d_, the exact same parameters are obtained in **B** as from fit of the concentration–response data in **A**.

Next, as a more complex case, let us consider fitting of the same type of response data (EC_50_), but with integration of occupancy (*K*_d_) data obtained from a different, independent assay in the same system. As an example, the same simulated response data as above was used (Fig. 3; log EC_50_s of − 6, − 7, and − 8), but with an additional set of *K*_d_s (log*K*_d_s of − 5.2, − 6.6, and − 6.7). Fit with SABRE can be considered consistent if adequate fit can be obtained with all *K*_d_s restricted to their experimental value and a single amplification parameter *γ* characterizing the system (pathway) as a whole. The corresponding fit for the present data is shown in Fig. 5A. Very good fit could be obtained with only four adjustable parameters (3*ε* + 1*γ*): > 98% of variability could be accounted for with a well-defined gain parameter: *r*^2^ = 0.984, *γ* = 21.1 ± 4.7. While this was done here with simulated data customized to illustrate such fit, examples of experimental data that can be fit with SABRE using a unified amplification parameter are also available. For example, contraction of isolated rat aorta data obtained for imidazoline type α-adrenoceptor agonists^11^, which are used as textbook illustration for possible mismatch between *f*_resp_ and *f*_occup_^38^, could be fitted very well with SABRE with the assumption of a single gain (amplification) parameter for data from five compounds (*γ* = 11.9 ± 2.0; *r*^2^ = 0.996; see Fig. 9 and Table S1 in Ref. ^16^).

Further, within the framework of SABRE one can not only fit such concentration-dependency data (Fig. 5A), but also directly link fractional response, *f*_resp_, to fractional occupancy, *f*_occup_^16^ (Fig. 5B). By using *f*_occup_ to replace [L] (see Supporting Information, Appendix 1),

23 $${f}_{occup}=\frac{\left[{LR}_{occup}\right]}{\left[{LR}_{max}\right]}=\frac{\left[L\right]}{\left[L\right]+{K}_{d}} \to \left[L\right]={K}_{d}\frac{{f}_{occup}}{\left(1-{f}_{occup}\right)}$$

one gets the corresponding function that can be used for direct fitting:

24 $${f}_{resp}=\frac{\varepsilon \gamma {f}_{occup}}{\varepsilon \left(\gamma -1\right){f}_{occup}+1}=\frac{\gamma }{\gamma -1}\frac{{f}_{occup}}{{f}_{occup}+\frac{1}{\varepsilon \left(\gamma -1\right)}}$$

Typically, binding data are used to generate *K*_d_ estimates (usually via a Cheng-Prusoff correction^39^). If these *K*_d_s are then used to calculate occupancy (*f*_occup_) at the [L] values were response data (*f*_resp_) are available, fit with the above equation provides the same parameters as obtained from direct fit of the concentration–response data. Illustration for the present data is provided in Fig. 5B. While models based on the two-parameter operational model (Eq. 4) can be used to fit the response data (with some known problems fitting full and close to full agonists), they have difficulties connecting the response to independently determined binding data (*K*_d_) as done here with SABRE. One exception is a three-parameter “special edition” extension of the operational model “with given *K*_d_ values” introduced by Rajagopal and Onaran for bias quantification that uses experimental *K*_d_ values as its *K*_D_^19,40^. In this model, *K*_D_s are not fitted, but replaced with experimental ones to constrain the regression. To achieve this, an additional scaling factor *α* (*r*_max_) needs to be introduced in Eq. 4 to allow a scalable *E*_max_:

25 $${E}_{/{E}_{max}}={f}_{resp}=\alpha \frac{\tau \left[L\right]}{\left(\tau +1\right)\left[L\right]+{K}_{D}}$$

The three parameters of this model, however, can be linked directly to those of the present model (see Supporting Information, Appendix 2) so that parameters obtained from fitting SABRE can be used to derive those of this model (*K*_d_, *ε*, *γ* → *K*_D_, *τ*, *α*):

26 $${K}_{D}={K}_{d} ;\,\,\tau =\varepsilon \left(\gamma -1\right);\,\,\alpha =\frac{\gamma }{\gamma -1}$$

Conversely, the parameters of this “special edition” operational model can be transformed into those of SABRE using (Appendix 2):

27 $${K}_{d}={K}_{D};\,\,\varepsilon =\tau \left(\alpha -1\right);\,\,\gamma =\frac{\alpha }{\alpha -1}$$

This highlights again an important advantage of the present model, namely that it clearly separates the ability of the ligand to activate the receptor (*ε*) from the pathway-specific (post-receptor) signal amplification (*γ*), while these two are intermixed in the *τ* “transducer ratio” (“coupling efficiency”) parameter of the operational model and its different variants [i.e., *τ* = *ε*(*γ*–1) here; Eq. 26]. Hence, from the perspective of the present framework, the *τ* parameter of the operational model mixes together ligand- and pathway-specific effects separated into *ε* and *γ* here (see Appendix 2). Note also that this “special edition” operational model (Eq. 25) is somewhat unusual as it formally allows an *E*_max_ larger than 100% by having *α* = *γ*/(*γ*–1) > 1.

### Responses assessed at different vantage points

**Figure 6:**
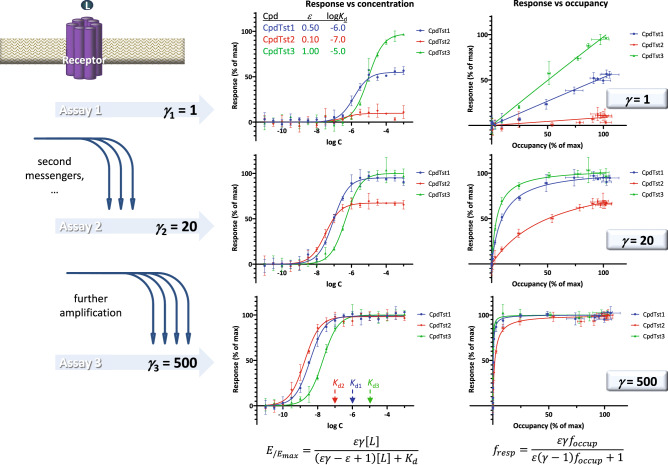
Multiple response data at different vantage points generated within the framework of the present SABRE model. Simulated data (symbols) for three different compounds were generated with the parameter values as shown (three compounds with log *K*_d_s of − 6, − 7, and − 5 and *ε*s of 0.5, 0.1, and 1.0 at three different readouts with *γ*s of 1, 20, and 500) and 5% random error; lines indicate corresponding model fits obtained with SABRE using a unified set of parameters (*n*_p_ = 9). Responses at the three different readout levels are shown as a function of both concentration (*f*_resp_ vs log *C*; middle column) and occupancy (*f*_resp_ vs *f*_occup_; rightmost column). Data were chosen so as to illustrate a possible nonintuitive shift in the order of potencies that can result from the strong downstream amplification of the response caused by a weak agonist: compound 2 (red; *ε* = 0.1) that generates the weakest response right after the receptor (assay 1, top) becomes the most potent one in assay 3 (bottom).

Next, let us consider the case of multiple responses measured at different downstream vantage points along the same pathway. This is of particular relevance for G-protein coupled receptors (GPCRs), known to involve signaling cascades with multiple second messengers. A set of such data generated within the framework of the present assumptions is shown in Fig. 6; they were chosen so as to illustrate a possible nonintuitive case (shift in the order of potencies) resulting from the intermingling of the effects caused by different efficacies at the receptor on one hand and increasing downstream signal amplification on the other. Simulated responses were generated for three hypothetical compounds of different affinities (log *K*_d_ of − 6, − 7, and − 5) and efficacies (*ε* of 0.5, 0.1, and 1.0) at three consecutive readout points of increasing amplifications (*γ* of 1, 20, and 500). At all three points, responses are shown as a function of both concentration (*f*_resp_ vs log *C*) and occupancy (*f*_resp_ vs *f*_occup_) (Fig. 6). Despite the unusual nature of the data, SABRE can provide integrative fit with a unified set of parameters (*n*_p_ = 9; 2 × 3 for the binding affinities and efficacies of the three agonists plus 3 for the amplifications of the three readout assays), not conceivable with operational model-based approaches that could only provide separate sets of *K*_D_s and *τ*s for each individual response (i.e., *n*_p_ = 18; 2 × 3 parameters of the three agonists for each of the 3 readout assays).

**Figure 7:**
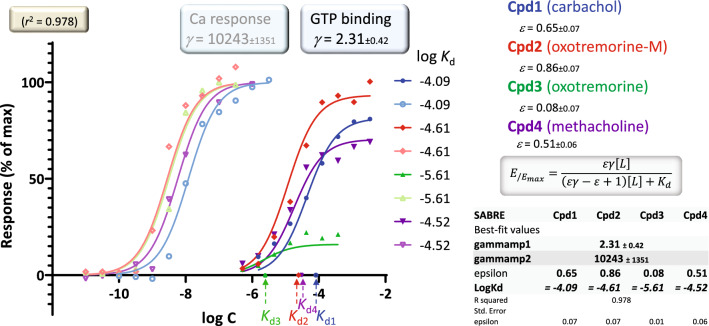
Fit of experimental response data obtained at different readout points with the present SABRE model. Data are for four muscarinic agonists with two different responses measured after M_3_ receptor activation (stimulation of GTP binding to Gα subunits /darker symbols/ and subsequent increase in intracellular Ca levels /lighter symbols/) and binding constants measured in equilibrium competition experiments with N-methyl-[^3^H]scopolamine (data after^12^). Experimental data are for carbachol (Cpd1, blue), oxotremorine-M (Cpd2, red), oxotremorine (Cpd3, green), and methacholine (Cpd4, purple). Fitting of *n*_d_ = 72 total data points with SABRE (lines) was done using the experimental *K*_d_ values (indicated on the *x* axis) and optimizing only *n*_p_ = 6 adjustable parameters: two *γ*s (one for each pathway) and four *ε*s (one for each test compound) as shown.

A further illustration is provided by fitting of a set of experimental data obtained from such consecutive responses (Fig. 7). They were measured at two consecutive vantage points after M_3_ muscarinic receptor activation by agonists such as carbachol, oxotremorine, oxotremorine-M, and methacholine: the stimulation of GTP binding to Gα subunits and the subsequent increase in intracellular calcium levels^12^. Receptor occupancy estimates are from log *K*_d_ values obtained from equilibrium competition experiments with *N*-methyl-[^3^H]scopolamine in the same work^12^. Unified fit of *n*_d_ = 72 data points for both responses from four compounds using SABRE with a single set of *ε* efficacies (one for each compound) and two gain parameters *γ* (one for each response) as adjustable parameters (*n*_p_ = 6) resulted in reasonable amplifications estimates (*γ* of 2.31 ± 0.42 and 10,243 ± 1,351 for the GTP and Ca readouts, respectively) and good overall fit (accounting for 97.8% of the variability in the data, *r*^2^ = 0.978) (Fig. 7). Because of a very strong (~ 10,000-fold) amplification in the second (Ca response) pathway, efficacy estimates are mainly defined by the first (GTP binding) response—obtained values and standard errors are summarized in Fig. 7. Such fittings can be considered consistent only if the unified single set of efficacies can provide acceptable fit at all the response levels considered for each compound including a full agonist. Nevertheless, unlike any other previous model, SABRE has the potential to connect response data assessed at different vantage points *k* (*E*_*k*_/*E*_*k*,max_) to affinities (log *K*_d_) and intrinsic efficacies (*ε*) for multiple compounds as long as reasonable overall fit can be obtained with unified gain parameters (*γ*_*k*_).

### Biased agonism

Finally, let us consider the application of the present model for quantification of biased agonism. It has been recognized since at least the 1990s that some receptors can engage multiple downstream signaling pathways simultaneously and activate them differentially in different tissues (e.g.,^41,42^). For such receptors, and in particular for GPCRs known to couple to several G proteins as well as β-arrestins, it is conceivable that certain ligands can show differences in their ability to activate these pathways—a phenomenon designated as biased agonism (stimulus bias, functional selectivity, or ligand directed signaling)^22,40,43–52^. For this to exist, there have to be (1) different active states of the receptor (i.e., R^*^, R^#^, etc.) that can preferentially initiate distinct downstream signals, (2) coupling proteins that differentially recognize these different active receptor states (e.g., G proteins, GPCR kinases, and β-arrestins), and (3) agonists capable to differentially stabilize these active states. Structural evidence is beginning to accumulate in support of this^53^.

**Figure 8:**
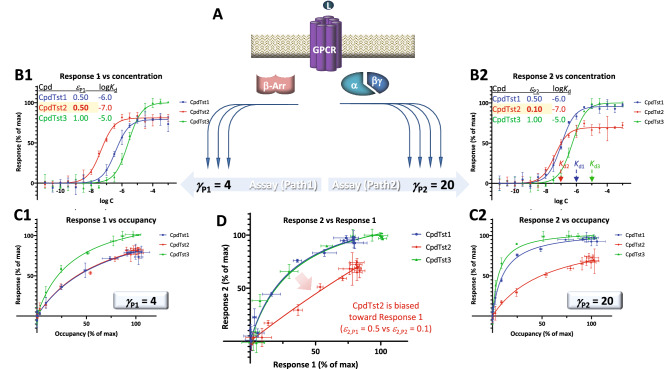
Illustration of biased agonism with response data for two different downstream pathways originating from the same receptor (**A**) generated with the assumption of the present SABRE model. Simulated data (symbols) for three hypothetical compounds were generated as before (Fig. 6) using the parameter values shown (CpdTst2 having two different efficacies *ε* for pathways P_1_ and P_2_ as highlighted in yellow; see text for details). Data for two pathways involving different signal amplifications (*γ*_P1_ = 4 left and *γ*_P2_ = 20 right) are shown as classic semi-log concentration–response curves (*f*_resp_ vs log *C*; **B1**, **B2**), fractional response versus occupancy curves (*f*_resp_ vs *f*_occup_; **C1**, **C2**), and a bias plot (*f*_resp1_ vs *f*_resp2_; **D**).

An illustration of such data generated within the framework of the present assumptions for two divergent pathways involving different signal amplifications and three hypothetical compounds of different efficacies is shown in Fig. 8. Corresponding data are shown as typical concentration–response curves (*f*_resp_ vs log *C*), response versus occupancy ones (*f*_resp_ vs *f*_occup_), and a typical bias plot used in such cases (i.e., relative response plot of *f*_resp1_ vs *f*_resp2_^44,54^). Data were generated for two different pathways (P_1_, P_2_) originating from the same receptor, but having different amplifications (*γ*_P1_ = 4 and *γ*_P2_ = 20) and three hypothetical compounds having different affinities (log *K*_d_s of − 6, − 7, and − 5) and efficacies *ε*. Two compounds (1 and 3) had the same efficacy for both pathways (*ε*_1,P1_ = *ε*_1,P2_ = 0.5 and *ε*_3,P1_ = *ε*_3,P2_ = 1.0), while one (2) had a higher efficacy for pathway 1 (*ε*_2,P1_ = 0.5, *ε*_2,P2_ = 0.1) generating a biased response as most evident in the bias plot of Fig. 8 (bottom row, center).

Because of the intermix of effects related to binding, receptor activation, and signal amplification, quantifying signaling bias is difficult, and it may not be achievable in many cases^19,55^. Current bias quantification methods typically rely on calculating ΔΔlog(*τ*/*K*_D_) or ΔΔlog(*E*_max_/EC_50_) versus a selected reference compound, e.g., a logarithmic bias factor is obtained as (log(*E*_max,P1,L_/EC_50,P1,L_)–log(*E*_max,P2,L_/EC_50,P2,L_)) − ((log(*E*_max,P1,Lref_/EC_50,P1,Lref_)–log(*E*_max,P2,Lref_/EC_50,P2,Lref_))^19,49^. This originated, in fact, from the concept of ratio of equiactive molar ratios, later termed intrinsic relative activity (RA_i_) introduced by Ehlert and co-workers, which is the ratio of *τ*/*K*_D_ fractions with the operational model and becomes that of *E*_max_/EC_50_s for a Hill slope of 1, i.e., (*E*_max,L_/EC_50,L_) / (*E*_max,Lref_/EC_50,Lref_)^56,57^. Because SABRE explicitly separates pathway amplification from ligand efficacy, it might allow a conceptually different approach, as discussed briefly before^16^. If data can be fitted with sufficiently well-defined gain parameters (*γ*_P*k*_, one for each pathway P_*k*_), then pathway-specific differences in amplification can be separated from those in ligand-specific efficacies, and these *ε*s can serve as cleaner indicators of bias, possibly even without predefined reference agonists. If receptor occupancy (*K*_d_) data are available, and SABRE can fit the data for each pathway adequately, one can calculate ligand efficacies for each pathway separately and compare them for indication of bias. If fitting can be done so that full or close to full agonists (*ε* = 1) are identified for each pathway, ligands that have efficacy ratios (*ε*_P*k*_/*ε*_P*l*_) significantly different from 1 can be considered as being biased agonists. Otherwise, *εγ* products need to be compared. There are methods to estimate efficacies with two-state models^58^; nevertheless, because SABRE is the first model that explicitly uses separate parameters for efficacy (*ε*) and amplification (*γ*), it can untangle these connections in a manner not possible either with direct empirical comparisons (e.g., *E*_max_/EC_50_) or with fitting of previous models, which intermingled these two effects within the same parameter (e.g., *τ*, *χ*).

**Figure 9:**
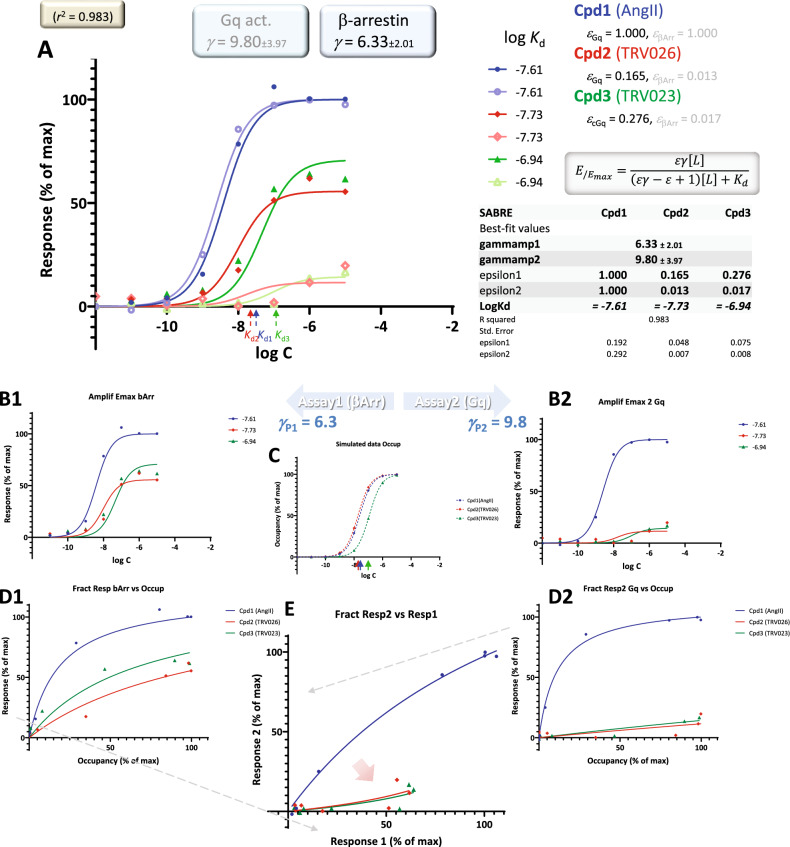
Fit of experimental response data obtained from divergent pathways with the present SABRE model. Data are for two responses measured along different downstream pathways (Gq-mediated inositol monophosphate increases and β-arrestin2 endocytosis) at the angiotensin II type 1 receptor (AT1R) (data after ^53^). Experimental data are for three agonists (angiotensin II, Cpd1, blue), TRV023 (Cpd2, red), and TRV026 (Cpd3, green) as indicated. (**A**) Fractional response versus log concentration data shown in a single composite graph. Fitting of *n*_d_ = 45 total data points with SABRE was done using the experimental *K*_d_ values and optimizing only *n*_p_ = 8 adjustable parameters (2 *γs* + 3 × 2 *ε*s) as shown. (**B**) Same response versus log concentration data, but shown in two separate graphs, one for each response. (**C**) Fractional occupancy versus log concentration data. The concentration dependency shown was calculated from the experimental *K*_d_ values with standard Hill-Langmuir equation. (**D**) Fractional response vs occupancy data. Same data as in A, just shown as a function of calculated fractional receptor occupancy, not log concentration. (**E**) Bias (relative response) plot showing one fractional response as a function of the other. Compound 1 (AngII) is a balanced (nonbiased) agonist, the curvature is due to the slightly different amplifications for the two responses. Cpd2 and 3 are significantly biased and they elicit a stronger response along pathway 1 (β-arrestin) than pathway 2 (Gq).

To illustrate the process of bias detection with the present model, fit was done on recent experimental data obtained at the angiotensin II type 1 receptor (AT1R): two responses (Gq-mediated inositol monophosphate increases and β-arrestin2 endocytosis) generated with three agonists (angiotensin II, TRV023, and TRV026)^53^. Occupancies were calculated from experimental *K*_d_ values measured in the same work in equilibrium competition radioligand binding assays with [^3^H]-olmesartan. Fitting of *n*_d_ = 3 × (7 + 8) = 45 total data points could be done with only *n*_p_ = 8 adjustable parameters (two *γ* gain parameters, one for each pathway, and six *ε* efficacy parameters, two for each of the three test compounds). Note that currently, such simultaneous fit with the Prism implementation of SABRE is limited to eight data sets (i.e., two pathways and four compounds). It resulted in good fit (*r*^2^ = 0.983) with consistent and well-defined gain parameters for both pathways (*γ*_P1_ = 6.33 ± 2.01 and *γ*_P2_ = 9.80 ± 3.97). Obtained efficacies (Fig. 9) indicate angiotensin II (AngII, Cpd1) as a balanced full agonists (*ε*_1,P1_ = *ε*_1,P2_ = 1.0), but TRV026 and TRV023 as strong β-arrestin biased partial agonists that have significantly different efficacies for the two pathways (*ε*_2,P1_ = 0.165 ± 0.048, *ε*_2,P2_ = 0.013 ± 0.007; *ε*_3,P1_ = 0.276 ± 0.075, *ε*_3,P2_ = 0.017 ± 0.008). These indicate about 15-fold differences in efficacies (12.6 ± 7.2 and 16.5 ± 9.2 for TRV026 and TRV023, respectively), a bit less, but in general overall agreement with the approximately 100-fold bias suggested by the bias factors calculated in the original work from ΔΔ(*E*_max_/EC_50_)^53^. Because both activities were measured at the same ligand concentrations, a bias plot showing one response as a function of the other could also be constructed here easily, it is shown together with other graphs comparing fractional responses and occupancies in Fig. 9.

If binding data are lacking (no experimental *K*_d_s), which is the case for most biased agonism studies published so far, one can still use the present approach, but with fitted *K*_d_s. This should be done by enforcing a single unified *K*_d_ for each ligand to minimize the number of adjustable parameters *n*_p_. Nevertheless, even with this restriction, due to the more limited amount and nature of data, it typically becomes difficult to obtain well-defined parameters and a clear bias quantification. For example, for the data of Fig. 9, the fitting becomes ambiguous and none of the amplification or efficacy parameters can be obtained with well-defined values. The efficacy ratios for TRV026 and TRV023 remain about the same as before, but the standard errors are much wider than the values themselves (13.9 ± 15,285 and 17.6 ± 19,365). This is not surprising as the number of data points per adjustable parameters, *n*_d_/*n*_p_, here is only 4.1 (45/11) compared to 5.6 (45/8) before, which was much closer to the desired range of 5–10 needed for adequate fitting^5,33,34^. Accordingly, to improve the quantitative assessment of multiple responses and biased agonism, it is suggested to include experimental assessment of binding in the same system where response is measured whenever possible.

Finally, if needed, within the framework of SABRE one can directly fit bias plot^44,54^ (i.e., relative response) data that show one response as a function of another at the same ligand concentration (e.g., Figs. 8D and 9E). The function directly connecting fractional responses *f*_resp,P1_ and *f*_resp,P2_ generated along different pathways P_*k*_ at the same ligand concentrations [L] (hence, at the same *f*_occup_) can be expressed as^16^:

28 $${f}_{resp,P2}=\frac{{\varepsilon }_{P2}{\gamma }_{P2}{f}_{resp,P1}}{{\varepsilon }_{P1}{\gamma }_{P1}+\left[{\varepsilon }_{P2}\left({\gamma }_{P2}-1\right)-{\varepsilon }_{P1}\left({\gamma }_{P1}-1\right)\right]{f}_{resp,P1}}$$

Accordingly, pathways that have different amplifications (*γ*_P1_ ≠ *γ*_P2_) lead to relative response plots that are curvilinear even for balanced ligands L_*i*_ that do not show bias (i.e., *ε*_*i*,P1_ = *ε*_*i*,P2_):

29 $${f}_{resp,P2}=\frac{{\gamma }_{P2}{f}_{resp,P1}}{\left({\gamma }_{P2}-{\gamma }_{P1}\right){f}_{resp,P1}+{\gamma }_{P1}}=\frac{{\gamma }_{P2}}{\left({\gamma }_{P2}-{\gamma }_{P1}\right)}\frac{{f}_{resp,P1}}{{f}_{resp,P1}+\frac{{\gamma }_{P1}}{\left({\gamma }_{P2}-{\gamma }_{P1}\right)}}$$

While *K*_d_s are not needed for this type of comparison, quantitative fitting (Eq. 28) is likely to be ambiguous due to lack of sufficient data points per adjustable parameter. For example, for the case shown in Fig. 9, *n*_d_/*n*_p_ is only 21/8 = 2.6 resulting in very badly defined *ε* ratios. Nevertheless, bias plots can still be useful as they might allow identification of differences among ligands that are less evident in concentration–response or response vs occupancy plots (e.g., Fig. 8). However, both responses might be difficult to obtain at the same ligand concentration [L] and strong curvatures might confound assessments, especially in cases where pathways have considerably different amplifications.

## Conclusion

In conclusion, SABRE is a general two-state ensemble receptor model incorporating a corresponding quantitative form that can fit complex fractional response versus occupancy data. Contrary to previous models, it not only has more intuitive parameters that can be related directly to binding affinity (*K*_d_), activation efficacy (*ε*), and signal amplification/gain (*γ*), but it can incorporate experimental *K*_d_ values. Notably, it provides a unified framework to fit both complex and simple receptor data, as by constraining its parameters, the general equation (Eq. 5) can be converted into consecutively nested simplified models all the way down to the one-parameter Clark equation (Fig. 2).

## Supplementary information

Supplementary file 1

Supplementary file 2

## Data Availability

Data used for illustrations of model fit are either simulated data generated as described or reproduced from previous publications as indicated in the corresponding figures. The datasets generated and/or analyzed and a GraphPad Prism file with the implementation of the model discussed here are available from the corresponding author upon reasonable requests.

## Abbreviations

GPCR: G-protein coupled receptors
SABRE: Present model (with parameters for Signal Amplification, Binding affinity, and Receptor activation Efficacy)
