# Supplementary material for: A single unified model for fitting simple to complex receptor response data
Source: Sci Rep. 2020 Aug 7;10:13386. doi: 10.1038/s41598-020-70220-w (PMC7414914; doi:10.1038/s41598-020-70220-w)

SUPPLEMENTARY INFORMATION

# A Single Unified Model for Fitting Simple to Complex Receptor Response Data


*Peter Buchwald*[*]

Department of Molecular and Cellular Pharmacology and Diabetes Research Institute, Miller School of Medicine, University of Miami, Miami, FL, USA


- Appendix 1. Derivation of the Hill-type extension of the present SABRE model
- Appendix 2. Connection between the parameters of the present model and those of the operational model and its extension
- Appendix 3. Model implementation (GraphPad Prism)
- Prism file with implementation (SABRE_Demo_SciRep_v01.pzfx)

**APPENDICES**

## Appendix 1. Derivation of the Hill-type extension of the present SABRE model

As before [16], to obtain the full quantitative form corresponding to the most general version of the present model , we will express the fractional response, $f_{resp} = E/E_{max}$, first as a function of activated receptors, $f_{act}$, and then link it via the fractional occupancy, $f_{occup}$ to ligand concentration [L] while eliminating the unknown receptor concentrations. Binding is characterized by the equilibrium dissociation constant $K_d$ that represents an ensemble average for all active and inactive forms:

$$K_d = \frac{[L]([R]+[R^*])}{([LR]+[LR^*])} \quad \text{(A1.1)}$$

There are two efficacies: one an intrinsic, ligand characteristic (for a given receptor)

$$\varepsilon = \frac{[LR^*]}{[LR]+[LR^*]} \quad \text{(A1.2)}$$

and another a receptor characteristic baseline efficacy

$$\varepsilon_{R_0} = \frac{[R^*]}{[R]+[R^*]} \quad \text{(A1.3)}$$

With all these, the fraction of occupied receptors, $f_{occup}$, is

$$f_{occup} = \frac{[LR_{occup}]}{[LR_{max}]} = \frac{([LR]+[LR^*])}{[R_{tot}]} = \frac{([LR]+[LR^*])}{([R]+[R^*]+[LR]+[LR^*])} = \frac{\frac{[L]([R]+[R^*])}{K_d}}{([R]+[R^*])+\frac{[L]([R]+[R^*])}{K_d}} = \frac{[L]}{[L]+K_d} \quad \text{(A1.4)}$$

Note that $f_{occup}$ is only a function of [L] and $K_d$, it is independent of $\varepsilon$ and $\varepsilon_{R0}$, a consequence of defining $K_d$ as an ensemble characteristic for ligand-bound and ligand-free receptors irrespective of their activation status. The fraction of active receptors, $f_{act}$,:

$$f_{act} = \frac{([R^*]+[LR^*])}{[LR_{max}]} = \frac{([R^*]+[LR^*])}{([R]+[R^*]+[LR]+[LR^*])} == \frac{\varepsilon_{R0}([R]+[R^*])+\varepsilon([LR]+[LR^*])}{[R]+[R^*]+[LR]+[LR^*]} = \frac{\varepsilon_{R0}([R]+[R^*])+\varepsilon\frac{[L]}{K_d}([R]+[R^*])}{([R]+[R^*])+\frac{[L]}{K_d}([R]+[R^*])} =$$

$$\frac{\varepsilon_{R0}K_d+\varepsilon[L]}{[L]+K_d} \quad \text{(A1.5)}$$

Or to link it to $f_{occup}$ from above (eq. A1.4):

$$f_{act} = \varepsilon_{R0}\frac{K_d}{[L]+K_d} + \varepsilon\frac{[L]}{[L]+K_d} = \varepsilon_{R0}\left(1 - f_{occup}\right) + \varepsilon f_{occup} \quad \text{(A1.6)}$$

This translates into fractional response via a hyperbolic transduction (amplification) function that uses an odds-ratio type transform as input:

$$f_{resp} = E_{/E_{max}} = \frac{\Lambda}{\Lambda+\gamma^{-1}};\ \Lambda = \frac{f_{act}}{1-f_{act}} \quad \text{(A1.7)}$$

So that

$$f_{resp} = E_{/E_{max}} = \frac{\Lambda}{\Lambda+\gamma^{-1}} = \frac{\frac{f_{act}}{1-f_{act}}}{\frac{f_{act}}{1-f_{act}}+\frac{1}{\gamma}} = \frac{f_{act}\gamma}{f_{act}(\gamma-1)+1} \qquad \text{(A1.8)}$$

Hill type response can be introduced for the shape of the response (by replacing $\Lambda$ with $\Lambda^m$ to allow a more general type of amplification function) or for the shape of the binding (by replacing [L] with $[L]^n$ to allow for more general cooperative binding). Here, only the second version will be explored:

$$f_{occup} = \frac{[L]^n}{[L]^n+K_d^n} \qquad \text{(A1.9)}$$

and hence

$$f_{act} = \varepsilon_{R0}\left(1-f_{occup}\right) + \varepsilon f_{occup} = \varepsilon_{R0}\frac{K_d^n}{[L]^n+K_d^n} + \varepsilon\frac{[L]^n}{[L]^n+K_d^n} \qquad \text{(A1.10)}$$

This way, the fractional response becomes:

$$f_{resp} = \frac{f_{act}\gamma}{f_{act}(\gamma-1)+1} = \frac{\gamma\varepsilon\frac{[L]^n}{[L]^n+K_d^n}+\gamma\varepsilon_{R0}\frac{K_d^n}{[L]^n+K_d^n}}{(\gamma-1)\varepsilon\frac{[L]^n}{[L]^n+K_d^n}+(\gamma-1)\varepsilon_{R0}\frac{K_d^n}{[L]^n+K_d^n}+1} = \frac{\varepsilon\gamma[L]^n+\varepsilon_{R0}\gamma K_d^n}{(\varepsilon\gamma-\varepsilon+1)[L]^n+(\varepsilon_{R0}\gamma-\varepsilon_{R0}+1)K_d^n} \qquad \text{(A1.11)}$$

Hence, this Hill-type extension of the most general form of the present model that includes constitutive activity is a straightforward generalization introducing the Hill-coefficients:

$$E_{/E_{max}} = \frac{\varepsilon\gamma[L]^n+\varepsilon_{R0}\gamma K_d^n}{(\varepsilon\gamma-\varepsilon+1)[L]^n+(\varepsilon_{R0}\gamma-\varepsilon_{R0}+1)K_d^n} \qquad \text{(A1.12)}$$

In case of no constitutive activity ($\varepsilon_{R0}$=1), this simplifies to:

$$E_{/E_{max}} = \frac{\varepsilon\gamma[L]^n}{(\varepsilon\gamma-\varepsilon+1)[L]^n+K_d^n} \qquad \text{(A1.13)}$$

## Appendix 2. Connection between the parameters of the present model and those of the operational model and its extension

The original operational (Black & Leff) model [17] is a two parameter model whose parameters are denoted here as $\tau$ and $K_D$:

$$E/E_{max} = \frac{\tau[L]}{(\tau+1)[L]+K_D} = \frac{\tau}{(\tau+1)}\frac{[L]}{[L]+\frac{K_D}{(\tau+1)}} \quad \text{(A2.1)}$$

This model uses $K_D$ as a fitted parameter and cannot accommodate experimental $K_d$s. However, a three-parameter "special edition" extension of the operational model "with given $K_d$ values" has been introduced by Rajagopal and Onaran for bias quantification. This uses experimental $K_d$ values as its $K_D$ to constrain the regression; this requires introduction of one additional parameter ($\alpha$) and allows a scalable $E_{max}$ [19,40]:

$$E/E_{max} = \alpha\frac{\tau[L]}{(\tau+1)[L]+K_D} = \frac{\alpha\tau}{(\tau+1)}\frac{[L]}{[L]+\frac{K_D}{(\tau+1)}} \quad \text{(A2.2)}$$

Its three parameters ($\tau$, $K_D$, $\alpha$) can be directly connected to those of the three-parameter version of the present SABRE model ($\varepsilon$, $K_d$, $\gamma$; no constitutive activity, $\varepsilon_{R0}$=0)

$$E/E_{max} = \frac{\varepsilon\gamma[L]}{(\varepsilon\gamma-\varepsilon+1)[L]+K_d} = \frac{\varepsilon\gamma}{(\varepsilon\gamma-\varepsilon+1)}\frac{[L]}{[L]+\frac{K_d}{(\varepsilon\gamma-\varepsilon+1)}} \quad \text{(A2.3)}$$

Comparing the right-hand rearranged form of the above two equations (A2.2 and A2.3), equivalence is obtained if

$$K_D = K_d;\ \tau = \varepsilon(\gamma-1);\ \alpha = \frac{\gamma}{\gamma-1} \quad \text{(A2.4)}$$

Hence, with these substitutions, parameters obtained from fitting SABRE can be used to derive those of this operational model "with given $K_d$ values" ($K_d$, $\varepsilon$, $\gamma \rightarrow K_D$, $\tau$, $\alpha$). Conversely, the parameters of SABRE can be obtained from those of this "special edition" operational model ($K_D$, $\tau$, $\alpha \rightarrow K_d$, $\varepsilon$, $\gamma$) via:

$$K_d = K_D;\ \varepsilon = \tau(\alpha-1);\ \gamma = \frac{\alpha}{\alpha-1} \quad \text{(A2.5)}$$

In details:

$$E/E_{max} = \frac{\varepsilon\gamma[L]}{(\varepsilon\gamma-\varepsilon+1)[L]+K_d} = \frac{\tau(\alpha-1)\cdot\frac{\alpha}{(\alpha-1)}[L]}{\left(\tau(\alpha-1)\cdot\frac{\alpha}{(\alpha-1)}-\tau(\alpha-1)+1\right)[L]+K_d} = \alpha\frac{\tau[L]}{(\tau+1)[L]+K_d} \quad \textit{(A2.6)}$$

This highlights again an important advantage of the present SABRE model, namely that it clearly separates the ability of the ligand to activate the receptor ($\varepsilon$) from the pathway-specific signal amplification ($\gamma$) while these two are intermixed in the "transducer ratio" or "coupling efficiency"

parameter of receptor models based on the operational model, i.e., $\tau = \varepsilon(\gamma-1)$ here. In fact, this separation achieves exactly the one desired at the introduction of this model into two components as $\tau=\tau^*\varepsilon$ (with $\tau^*$ intended to account "for the amplification inherent to the downstream signaling pathway that is the same for all ligands in the same assay") [40] since in light of eq. A2.4 $\tau^*=(\gamma-1)$.

Incidentally, the parameters of the present model can also be linked to those of the original two-parameter operational model (eq. A2.1). Comparing the two models in their rearranged form (right-hand forms of eq. A2.1 and A2.3), they can be identical for all [L] values if both the pre-fraction multiplication factors and the fractions in the denominators are equal, i.e.:

$$\frac{K_d}{\varepsilon\gamma-\varepsilon+1} = \frac{K_D}{\tau+1} \;\&\; \frac{\varepsilon\gamma}{\varepsilon\gamma-\varepsilon+1} = \frac{\tau}{\tau+1} \tag{A2.7}$$

Solving this for $K_D$ and $\tau$ reveals that the two parameters of the operational model can be obtained from those of SABRE (fitted with a fixed $\gamma$ but adjustable $K_d$s for equivalent parametrization) via the following interconversion ($K_d$, $\varepsilon$, $\gamma \rightarrow K_D$, $\tau$):

$$K_D = \frac{K_d}{1-\varepsilon};\; \tau = \gamma\frac{\varepsilon}{1-\varepsilon} \tag{A2.8}$$

Again, this not only allows conversion, but also highlights a known problem of the operational model, namely that for close to full agonists (where $\varepsilon$ approaches 1), both $\tau$ and $K_D$ become unreasonably large and cannot be determined independently in well-defined manner. For true full agonists, $\tau$ becomes infinity, one of the reasons why this model cannot be converted back to simplified forms. Because of identifiability issues during regression of the operational model, only the "transduction coefficient" $\tau/K_D$ can be estimated precisely [18,19], and this is used most often for comparisons. From eq. A2.8, this is equivalent with $\varepsilon\gamma/K_d$.

Conversely, two of the three parameters of SABRE can be obtained from those of the two-parameter operational (Black & Leff) model ($K_D$, $\tau \rightarrow K_d$, $\varepsilon$, $\gamma$) via:

$$K_d = K_D\frac{\gamma}{\tau+\gamma};\; \varepsilon = \frac{\tau}{\tau+\gamma} \tag{A2.9}$$

Equivalent parametrization leaves the value of $\gamma$ undefined and it has to be set to a fixed value during fitting (e.g., $\gamma$=1 can be used assuming no amplification) as it simplifies out during the conversion:

$$E_{/E_{max}} = \frac{\varepsilon\gamma[L]}{(\varepsilon\gamma-\varepsilon+1)[L]+K_d} = \frac{\frac{\tau}{\tau+\gamma}\gamma[L]}{\left(\frac{\tau}{\tau+\gamma}\gamma-\frac{\tau}{\tau+\gamma}+1\right)[L]+K_D\frac{\gamma}{\tau+\gamma}} = \frac{\tau[L]}{(\tau+1)[L]+K_D} \tag{A2.10}$$

## Appendix 3. Model implementation (GraphPad Prism)

Equation used (user-defined equation in Prism):

Y=100*(epsilon*gammamp * (10^X)^n + epsilonR0*gammamp * (10^LogKd)^n)/
((epsilon*gammamp + 1 - epsilon) * (10^X)^n + (epsilonR0*gammamp + 1 - epsilonR0) * (10^LogKd)^n)

Parameter description:

| | | |
|---|---|---|
| X: | Log of dose or concentration | (corresponding to [L] here, eq. 5, Figure 2) |
| Y: | Normalized response, from 0 to 100 | (corresponding to $f_{resp}=E/E_{max}$, eq. 5) |
| logKd: | Logarithm of dissociation constant (conc.) | (corresponding to log $K_d$ here, eq. 5) |
| epsilon: | Efficiency (intrinsic, ligand) | (corresponding to $\varepsilon$ here, eq. 5) |
| gammmamp: | Gain (amplification) in new model | (corresponding to $\gamma$ here, eq. 5) |
| epsilonR0: | Efficiency basal (constitutive, receptor) | (corresponding to $\varepsilon_{R0}$ here, eq. 5) |
| n: | Hill slope | (corresponding to $n$ here, eq. 5) |

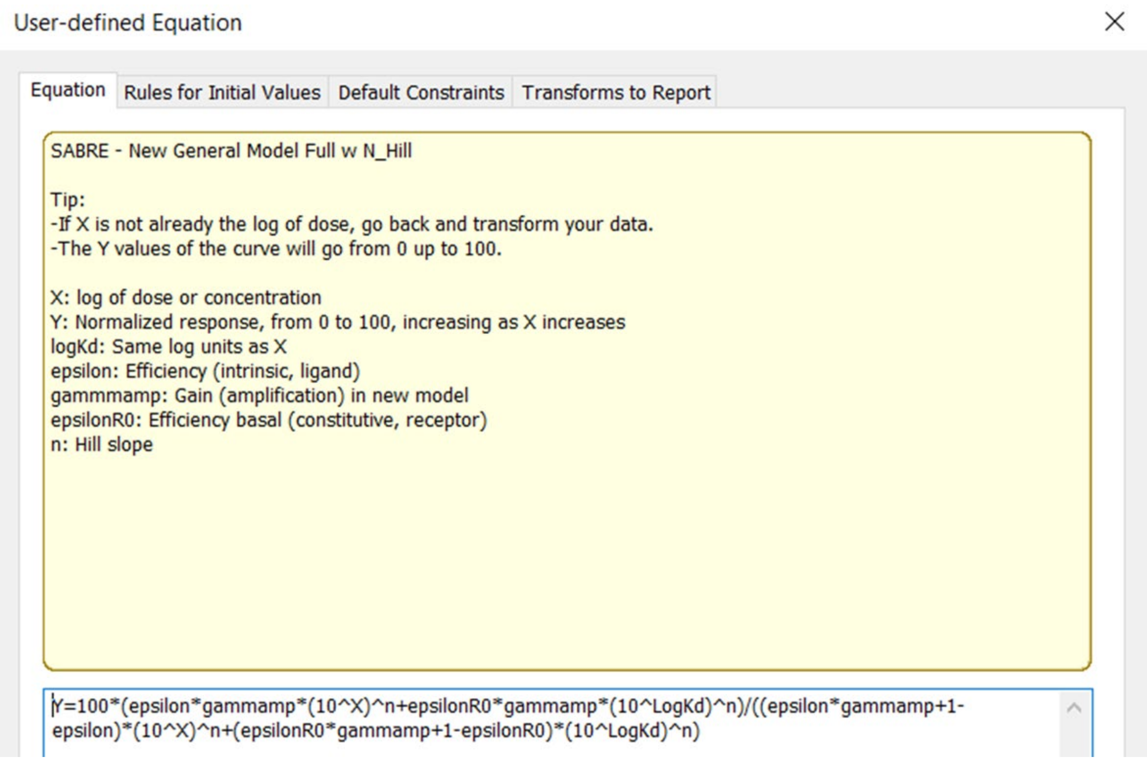

Typical constrain:

Parameters: Nonlinear Regression

Model | Method | Compare | Constrain | Initial values | Range | Output | Confidence | Diagnostics | Flag

| Parameter Name | Constraint Type | Value | Hook |
|---|---|---|---|
| epsilon | Must be between zero and | 1.00001 | |
| gammamp | Shared, and must be greater than | 1 | |
| epsilonR0 | Constant equal to | 0 | |
| LogKd | Data set constant (from column title) | | |

Sample output (results) and graph:

| | Nonlin fit<br>Table of results | A<br>-6.7 | B<br>-6.6 | C<br>-5.2 | D<br>Global (shared) |
|---|---|---|---|---|---|
| 1 | **SABRE - New General Moc** | | | | |
| 2 | **Best-fit values** | | | | |
| 3 | epsilon | 1.000 | 0.06594 | 0.2911 | |
| 4 | gammamp | 21.06 | 21.06 | 21.06 | 21.06 |
| 5 | n | = 1.000 | = 1.000 | = 1.000 | |
| 6 | epsilonR0 | = 0.000 | = 0.000 | = 0.000 | |
| 7 | LogKd | = -6.700 | = -6.600 | = -5.200 | |
| 8 | Kd | = 1.995e-007 | = 2.512e-007 | = 6.310e-006 | |
| 9 | **Std. Error** | | | | |
| 10 | epsilon | 0.1970 | 0.01423 | 0.05936 | |
| 11 | gammamp | 4.736 | 4.736 | 4.736 | 4.736 |
| 12 | **95% CI (profile likelihood)** | | | | |
| 13 | epsilon | 0.6044 to ??? | 0.03742 to 0.07565 | 0.1691 to 0.3383 | |
| 14 | gammamp | 18.58 to 37.91 | 18.58 to 37.91 | 18.58 to 37.91 | 18.58 to 37.91 |
| 15 | **Goodness of Fit** | | | | |
| 16 | Degrees of Freedom | | | | 149 |
| 17 | R squared | 0.9859 | 0.9653 | 0.9854 | 0.9843 |
| 18 | Sum of Squares | 1394 | 1331 | 1146 | 3871 |
| 19 | Sy.x | | | | 5.097 |
| 20 | **Constraints** | | | | |
| 21 | epsilon | 0 < epsilon < 1.00001 | 0 < epsilon < 1.00001 | 0 < epsilon < 1.00001 | |
| 22 | gammamp | gammamp is shared | gammamp is shared | gammamp is shared | |
| 23 | n | n = 1 | n = 1 | n = 1 | |
| 24 | epsilonR0 | epsilonR0 = 0 | epsilonR0 = 0 | epsilonR0 = 0 | |
| 25 | LogKd | LogKd = -6.7 | LogKd = -6.6 | LogKd = -5.2 | |

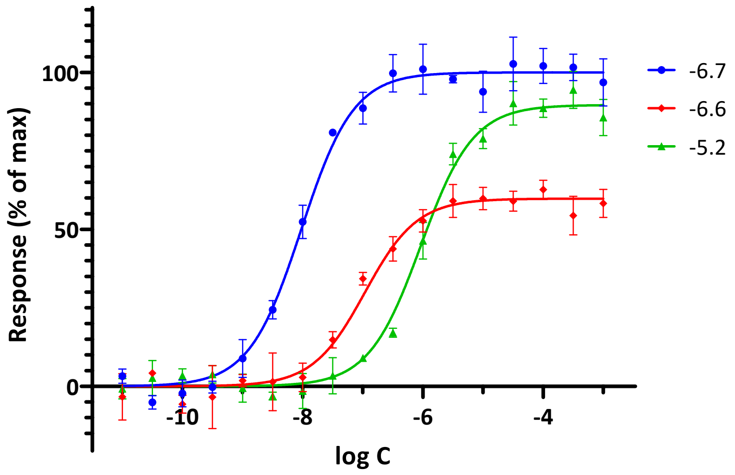

Supplement: Supplementary file 1 [file 41598_2020_70220_MOESM1_ESM.pdf]
